# Worldwide Experience of Breast Implant-Associated Large Cell Lymphoma (BIA-ALCL): Expert Panel and Roundtable Discussion

**DOI:** 10.1093/asjof/ojz020

**Published:** 2019-07-18

**Authors:** Jeffrey M Kenkel, Mark Magnusson, Nigel S G Mercer, William P Adams

**Affiliations:** 1 Department of Plastic Surgery, UT Southwestern Medical Center, Dallas, TX; 2 Griffith University, Southport, Queensland, Australia; 3 Department of Plastic Surgery, and Program Director of the Aesthetic Surgery Fellowship at UTSW, University of Texas Southwestern, Dallas, TX

We are pleased to present this roundtable discussion, recorded live at The Aesthetic Meeting 2019 in New Orleans, featuring the most up-to-date, evidence-based knowledge on breast implant-associated large cell lymphoma (BIA-ALCL) from a panel of worldwide experts (a transcript of the discussion is available online as Supplemental Material at www.asjopenforum.com). The panelists share updates on the entity from their region and also their experience with BIA-ALCL. The panelists have been published extensively on this subject^1-6^ and were selected for their expertise, worldwide perspective, and history of patient safety advocacy. It’s important to note that research in this field is rapidly evolving^7^ and we encourage you to stay current by reading the journals in our field and attending meetings featuring BIA-ALCL panels and discussions.

## Disclosures

Dr Kenkel received an educational grant from Galderma for the anatomic laboratory; and research grants from Bellus Medical and Venus Concept. Dr Magnusson received no funding support for this presentation; however, he is a current advisor to Allergan and has a past educational association with Mentor. Mr Mercer was a member of the Allergan Expert Forum from 2011 to 2012. Dr Adams declared no potential conflicts of interest with respect to the research, authorship, and publication of this article.

## Funding

The authors received no financial support for the research, authorship, and publication of this article.

## Supplementary Material

ojz020_suppl_Supplementary_Material
