# Supplementary material for: Worldwide Experience of Breast Implant-Associated Large Cell Lymphoma (BIA-ALCL): Expert Panel and Roundtable Discussion
Source: Aesthet Surg J Open Forum. 2019 Jul 18;1(3):ojz020. doi: 10.1093/asjof/ojz020 (PMC7780434; doi:10.1093/asjof/ojz020)
Supplement: ojz020_suppl_Supplementary_Material [file ojz020_suppl_supplementary_material.docx]

Jeffrey M. Kenkel, MD: Hi, I’m Jeff Kenkel, I’m associate editor of the *Aesthetic Surgery Journal*. And along with the Plastic Surgery Channel, I really want welcome you to this very educational session on *Breast Implant-Associated Anaplastic Large Cell Lymphoma*, or ALCL. And we’ve got three of the world’s experts here to kind of share not only their knowledge about the entity but also their regional experience and kind of update us on where we are worldwide.

So, we’ve got Bill Adams from Dallas, Texas, who’s going to tell us a little bit about the US experience; Nigel Mercer from Bristol, England, is going to talk a little bit about Europe and the UK; and then, finally, Mark Magnusson from Queensland, Australia, can kind of update us on where we are in Australia.

So, Bill, why don’t you start off. Talk to us a little bit about where we are in the US with this entity. And then, maybe update us on the FDA panel hearings. You were intimately involved with those.

William P. Adams, Jr., MD: Yes, absolutely. So, Jeff, having not known anything about this disease 10 years ago, I think we’ve learned a wealth of information. And the good thing is that we actually know quite a bit about what the cause is and how to treat it properly when it’s diagnosed. So, those are good things for patients. But in the US, we have a profile registry where those cases are tracked, and basically there’s about 164 confirmed cases in the profile registry. And in the United States, we’ve had five deaths from breast implant ALCL.

This was, obviously, talked about a lot at the FDA committee meetings, in March. And, there’s certainly women who have had abnormal presentations to this and had a lot of issues and bad outcomes. But the vast majority of people, in the United States, do present with a seroma or a late seroma, and they can be diagnosed by sending a couple of simple tests, and then they’re treated with a capsulectomy.

And what was brought out of the meetings – some of the experts here on the (FDA) panel – is that you look at relative risks, even things like cancer drugs like aromatase and other things, they have known side effects. And some of those relative side effects that people deal with are far and above more common and more serious than some of the things you see with ALCL. So, at the end of the day, the FDA just came out on May 2^nd^ stating that even though this is associated with textured breast implants, they didn’t feel there was enough data or evidence to remove any of the textured implants or any of the macrotextured implants. And that was the most recent decision coming out of the U.S. regulatory body.

Kenkel: Nigel, can you update us on where you guys are in the UK?

Nigel Mercer, MD, FRCS: Well, about 300 cases, in Europe, to date. We’ve had about 40 cases in the U.K. and we’ve had one death associated with ALCL. So, we’re not seeing quite the same spectrum of disease, and that’s one of the interesting things – we seem to be seeing different things in different countries. We’re not seeing clustering around cities or around surgeons; we’re seeing an even spread associated with the numbers of implants sold into the market. We haven’t seen any definitely associated with only smooth implants, but we have to remember that we look at smooth implant, you can see through it, but at the cellular level, it isn’t smooth.

And I think all the countries are now agreeing that the texturing, how we grade textures internationally, is just a mess. And we need someone to come up quickly with a good way of designating that. But getting the message out is the big, important thing. It still remains a rare disease. We think it’s associated 1 in 24,000 implants in the UK; so, if someone’s had 2, that’s a risk of 1 in 12,000. And I know it’s different in Australia, and the Dutch information that’s coming through is slightly different as well.

But, we’ve only had 600 or so cases in the whole world, and that’s not really enough for us to know a great deal about the whys and wherefores. And the important thing is, for us, as surgeons, when we see patients who are having an augment of any reason, we’ve got to make sure the patient’s well informed, they have all their choices, they know what to look out for. And patients who have the implants in need to know they don’t need to panic, they just need to know what to look out for.

Kenkel: Mark, and the leadership in Australia and New Zealand have really worked pretty intently with the government, and you guys have really broken down the data, at least the data that you have, in a very nice way. Can you share with us kind of what you know right now?

Mark Magnusson, MD, BS, FRACS: Sure. Okay so, at the present, as you’ve said, we work with the Australian Breast Device Registry and the government, our Therapeutic Goods Association, which is our equivalent of the FDA. And at the present, we’ve got at least 90 cases; we’ve got others that we’re working up at the moment. And, about 70% of those are pure single implant cases, which is important. As Nigel indicated, there are certainly a large number of patients who have multiple implant sets, and it’s difficult to then know how to relate that to incidence.

But, what we’ve been able to do – because of the numbers – we have full implant history on our patients, except for one patient who had an implant maybe 20 years ago where we don’t know what that single implant is, but we know what their subsequent ones are. So we’ve got very clean dataset. Everyone is confirmed pathologically. So we’ve been able to do subset analysis because we’ve got to a significant number.

So, we certainly have been able to demonstrate that the different implants have a different relative risk. But, one of the important things is not overextending what we’re finding in our geography, with our patient subset, into other markets. Because, as Nigel’s clearly said, in the UK and in Europe, they’re not seeing quite the same patterns. And our data is based on information relating to three implant styles, one of which is no longer available. So, from that, there’s been certainly an extrapolation and interest.

And we have developed a theory – but again, at this point, it’s a theory – in terms of the etiology of the disease and the pathogenesis and how we go from breast implant to ALCL. And that relates to a number of points. The first is we’ve got an implant with bacteria on it. We’ve got a patient responding to that with an initial polyclonal immune response, inflammatory response. One of those cell lines may become monoclonal. But that doesn’t mean that’s going to become a lymphoma.

We then find that we’ve got genetic aberrations in these cells. The genetic aberration we’re talking about has a 1 in 10,000 risk in the normal population. And of the cell lines we’ve examined, they’ve all got an aberration affecting the function of this particular gene. So, there are certain genetic elements, and then we can get transformation. And those genetic elements may, in part, explain why we get differences in distribution, why worldwide there is only one Asian patient. And that includes Asian patients here, in America, and in our country, in Australia. So, it’s not just a lack of detection, it’s across the field.

So, as I said, I think we’ve got very good data, it’s giving us good indications of what we’re seeing. We also had, in our very first data we put out, three deaths. In the last four years, we’ve only had one further death. We are much better at detecting and at treating. And, while that certainly is a tragedy, I think one of the other things is we’re much better – despite 700 cases – there’s so much collaboration across the oceans, across the borders, that we’re really working out a lot of information quickly, but we’re not there yet.

Kenkel: Nigel, maybe you can comment. I think there’s a lot of confusion about what textured implants are available, particularly in Europe, and which ones have been banned. Can you kind of update us on what implants are currently not available, and how it relates to the CE mark and things like that?

Mercer: So, CE marking is a bit like the FDA approval. But actually, in Europe, it’s really about how something’s made; there’s no safety testing that goes with CE marking. And, what happened with one of the implants, one of the salt-loss technology implants, they lost their CE mark because their information and submission was not looked at ahead of the French regulators (ANSM) meeting earlier this year. And so, those implants are no longer sold into the market, and that happens to be the same implant that’s got the highest incidence – or one of the highest incidences, we should say – in the Australian data. So those have been off the market now for some time, and it’s not relating necessarily to ALCL, just the fact they haven’t got a CE mark anymore.

So, one of the issues because of CE marking is that if you can show equivalence of how something’s made, you can get a CE mark, and it’s sold into the market. So the British market, in particular, we’ve had loads of different implants, which hasn’t necessarily been the case in other countries. So the profile of the implant and what’s the disease relationship to that is different, as we’ve just been saying.

So, for example, the French have decided to ban, after their hearing, certain salt-loss technology macro- textured implants, but they haven’t banned them all. And the reason they haven’t banned them all is that some of them aren’t sold into that market directly, but they are still available within that market. So, what we’re very concerned about, in Europe, is there’s sort of a political knee-jerk reaction to something without there being significant science behind it.

The Dutch, on Friday of this last week, have said that according to their data, it looks as though it’s very strongly associated with the Biocell implant, as you said. They agree that it’s no longer available because it’s not CE marked, and so they don’t need to do any further action about that. But, for example, what the Dutch have said is that every patient who has a Biocell implant, in Holland, now needs to be contacted, to be told that they have the potential risk. And from the General Data Protection Regulations, in Europe, that is a nightmare, an absolute nightmare.

Adams: Yes, I think what’s critically important is that it is the wrong kneejerk reaction to say this is just about a certain implant or a certain class of implants, because what Mark said about this unification theory, there’s multiple things that need to be in place for this disease to develop. So, you need chronic inflammation, which we presume is probably bacteria. Textured implants are associated with it because they have a higher surface area, so that’s been shown scientifically to have much higher numbers of bacteria on those implants. You need some sort of genetic predisposition, and you need time – it takes eight to nine years for this disease, generally, to develop.

So, it’s all those things. It creates a false sense of security, I think, to suggest that we just take these implants off you’re not going to see the disease. And in fact, it gets into probably what we’ll talk about, too, is there may be some ways that we can reduce risk. Some people have looked at we should just not use textured implants. And I think that’s going too far, again, because there have been studies that have shown surgical technique, things that have minimized the bacterial load, things like that that we’ve known have reduced things like capsular contracture may reduce risk for this disease.

That’s not uniformly agreed upon amongst the plastic surgery community; even at this meeting, you see some disagreement on that. But one thing that I think is being overlooked is that the epidemiology when you see case clusters and the distribution of disease, the other theories they do not hold up to that. You will not see case clusters, if it was coming from, say, a particle on macrotextured implants, you would tend to see a lot more cases in places that use a lot of macrotextured implants, and you wouldn’t see this clustering of diseases. There are actually 17 case clusters globally with this, which really does indicate more of an infectious trigger. Again, the bacteria are probably not the only thing involved, but they are clearly a part of it.

Mercer: There are the competing theories and there is a lady in Cambridge University in England, who is doing some interesting work, looking at the immunology around it. So that we need to get all the people who are looking at this, and we need some state-sponsored research into this as well. I chair the panel who advises our MHRA, the same as your FDA, about this problem. The MHRA have said if we need to go to our Department of Health and say we actually need some money putting behind this. We need a monoclonal ALCL strain, which could then be grown on implants and actually get some good research done.

I’ve just done a literature review for a paper I presented a week ago and, out of the 400 papers or so, there is no science, there is just none out there. We have so many unknowns here, we just are wading around a bit.

Adams: Yes, I mean the indolence of the disease and the small number of cases, it’s a difficult thing. It may take many, many, many years to really maybe solve all the pieces of the puzzle. But the thing that we do know is that awareness is critical to diagnosing people when they present and treating them appropriately. There are a few outliers, but most of the bad outcomes, especially initially, were complications of chemotherapy or other types of therapies, or patients that just never had any intervention, they were placed on antibiotics. To my knowledge, globally, 100% of people who have been diagnosed promptly and treated have been cured.

Magnusson: And that’s important because our data brings that out as well. The majority of patients present in the early stage of disease; it’s not a rapidly progressive problem at that time. And if we look at those who present in the early stage of disease, which as Bill just said, is curable with the proper operation without radiotherapy and chemotherapy when it’s diagnosed at that point. And that’s more than 80% of the patients in our series. And one of the significant differences between that group and the late presentation/more advanced stage is duration of symptoms. So, it’s about awareness, and it’s about not missing the diagnosis.

Kenkel: So, we talked about awareness of the physicians of the entity but also the patients, and that’s just part of your informed consent process. So, Mark, we have a patient who we’re concerned. They have acute swelling, it’s X amount of years after their implant-based surgery, I mean, what do you do next?

Magnusson: Okay, so the very first thing is to confirm that that swelling relates to what we’re considering here in this disease, the most likely presentation is a delayed seroma. So that’s a collection of fluid around the implant that’s more than 12 months after insertion of implant. So, the gold standard of testing, at this point, would then to tap that fluid under radiological guidance – because, of course, we don’t want to damage the implant – and that fluid is removed, and it’s tested appropriately. We should be seeing anaplastic large cells. We should be able to test them with immunofluorescence for CD30, and these cells have CD30-positive, and they also have another marker, ALK-negative.

And this is a diagnostic process that we get out of that with immunofluorescence. And that allows us then to go right, we’ve got this disease process, the patient should be worked up further with either a PET/CT scan or MR or both. The MR’s going to give us a very clear picture of what’s happening locally in the disease, and PET/CT scan is going to help us pick up that small number of patients that do have metastatic disease or more significant spread. Then, as most of these patients are being managed at that early stage, working up for theater, and the operation is en bloc capsulectomy. At this point, in relation to breast implants, this is the only true indication of en bloc capsulectomy.

For those small number of patients who have more advanced disease, this disease is best managed in a multidisciplinary team. And that could include a breast surgeon, and certainly, all of these patients should have the inclusion of hematologist with understanding about this disease. Because this disease, it develops and is managed in a different way to other lymphomas. This is a surgical disease. You remove that disease, and we don’t need to consider chemotherapy and radiotherapy for early-stage disease managed properly, surgically. But, we still need follow-up and there will be those few patients who either have incomplete excision or more distant disease. And for them, the chemotherapy and potentially radiotherapy options will need to be considered.

Kenkel: So if I’m a plastic surgeon in one of your countries, tell me a little bit about registries and any other research opportunities. Bill, maybe you want to mention Marshall Kadin’s study. Is there any other way that we can get more information to the right people?

Adams: Yes, so as Mark alluded to, patient comes in with a seroma, you can confirm that with ultrasound. You’ll send it off for the screening test, which will be CD30 and cytology. Now the one thing, if a patient comes in, the majority of those patients don’t have breast implant ALCL, they have a late seroma that’s nonmalignant. Even there’s a subset of patients – about to 10 to 12% of late seromas are CD30-positive but negative histology or cytology, and they fall into a category as a nonmalignant but some sort of in-between form of this. But, the vast majority of people don’t have BIA-ALCL.

But, studying those early seromas are very important to our understanding of the disease. So Marshall Kadin, who actually has done a lot of work and is a renowned dermatopathologist, is studying early seromas. Most of the ones that we have sent have been benign. But he will study malignant seromas if you have that. And that information is on the Aesthetic Society’s website

[here](https://www.surgery.org/professionals). There’s even a FedEx number; you can package your seroma and send it to him. It’s actually been very helpful. He presented at this meeting and has shown that there are certain markers that are present in both benign and malignant seromas. He’s shown that the cytokine profiles in these seromas beyond CD30 are elevated in malignant seromas. And ultimately, this may help us diagnose patients quicker or better in the future.

Kenkel: Nigel, what about in your neck of the woods? Do you have a centralized registry that you want people to participate in?

Mercer: Well unfortunately, we had a breast implant registry which lost its government funding some years ago, and the new one, we’ve only just got permission now to have it as an opt-out registry. And that’s only been going for about four years now. So our numbers are low. All cases have to be reported to MHRA, and it’s absolutely essential that that happens and the treatment and diagnostic pathway is followed. And I think one of the important things, having an expert pathologist look at the cells is very important because CD30 cells do occur normally in some seromas, as Bill has just said.

So, what we’re saying to our surgeons and to the patients is we want double reporting. We want as many reports as possible on these, and we’ll sift out if we’ve got double reporting. But what we must not be doing is missing any of these cases. And with us, because it’s still relatively rare with us, we’re wondering whether or not we should have maybe two centers in the country who deal with the more advanced cases. Because we don’t want to have different units doing different things to different patients. And that would be the worry. We know how to treat these. The evidence that’s come out of MD Anderson really shows the type of immunotherapy that works for the more advanced cases, and the patients should be getting that rather than getting ordinary chemotherapy, which we know has got significant side effects.

Kenkel: So we’ve discussed textured surface implants, what about smooth implants? I know there was some discussion, at the FDA at the panel discussions, about smooth implants. Bill, what can you tell us about smooth implants and their risk?

Adams: Yes, people have had questions about that. The FDA has put out statements. We’ve actually talked to the FDA directly, and so we know what their input on this is. But, they’re just trying to basically report what their MAUDE database shows. What their statement says is that they know that most of the cases are associated with textured implants, but they are aware of a much smaller number of smooth implant cases. But, what they’re not coming out and saying, but what we know globally, that there’s never been a pure only/smooth only case in any registry or database to date.

Now, some of the cases are unknown, but right now, we’re not aware of any pure only smooth implant case. Now does that mean that you can absolutely not get it in a smooth implant? I don’t think that’s necessarily true. And at some point, there may be a smooth case, although some of the things we’ve talked about, you just may not be able to get a high enough bacterial load over six to eight years without the implant just being frankly infected. I think that’s some of the theories that talk about it. But, Nigel, you guys have just come out with a statement around that, too, right?

Mercer: Yeah, pretty much, because just because it hasn’t happened yet doesn’t mean it’s not going to happen in the future. Because at the cellular level, something that’s smooth still has got significant irregularities in it. So, MHRA has said in our current state of knowledge, we can’t say that every implant is safe, or we can’t say that any implant is safe. We just have to be watchful. And as we all know, particularly reconstructive patients, textured implants have got significant benefits over a smooth implant, particularly if you want to go for an anatomical implant.

So, the Dutch have felt, and as have we, that if we take a knee-jerk reaction, we’re going to really limit some of the reconstructive options that particularly the breast cancer patients may have. But of course, the patient needs to make the decision.

Kenkel: So, this has been a great discussion. I mean, we have representatives from all over the world talking about how we’re approaching this entity today. What’s in the future?

Magnusson: Okay so, certainly, there’s collaborative research that crosses borders and oceans. We collaborate with MD Anderson, we collaborate over on the East Coast in the States. And we need to set up these same sort of processes in other areas. One of the challenges is sharing biological products, and that’s a challenge – not insurmountable, but it’s still a challenge. Where we’re going with research, at the moment, we’re doing complete genomic sequencing on both the tumor cells and patients to see if we can break down what the apparent genetic influences may be, see if that helps.

We’re continuing to analyze the inflammatory markers around the tumor to see if we can look at all the variety of different theories that are there. Because the process of developing something like a lymphoma, we know that these cells respond to biological antigens – proteins, like gluten, bacteria, viruses, things like that. So, there is a precedent for that. There isn’t a precedent for some of the other things. But Suzanne Turner, who’s from Cambridge, is looking into some other aspects about whether the innate immune system is involved rather than the responsive immune system and things like that.

So we don’t have all these questions. And the only way we can get these answers is to share the data. I’m part of a research group, which is multinational, and it allows us to share these data, come up with theories, and then individually proceed along different lines of investigation so that when we come back and meet again, we’re answering more and more questions. But collaboration, we need to be doing this in a noncompetitive way because this is an important problem. Rare, but important.

Kenkel: I think without that collaboration, I mean, just think of the advances that we’ve made in just such a short time, and it’s really because of all the collaborative efforts. And without everybody’s unique experience, we wouldn’t have that information that we have to really give to our patients and to treat them the way that we need to treat them.

Well, thanks to the three of you. This has been extremely informative, and I think it’s great to be updated on where we are just around the globe with regard to the treatment. I know the Aesthetic Society really is at the forefront and really trying to lead the way with regard to disseminating information to its members and helping explore not only treatment but also prevention and understanding how this whole thing occurs.

There’s also been a lot of really good articles that have been published in great journals like the *Aesthetic Surgery Journal*. So, thank you all for taking the time, and we look forward to an update next year.

Adams: Absolutely, thank you.

Magnusson: Thanks. Thanks, Jeff.

Mercer: Pleasure, thank you very much.
